# Pro-angiogenic changes of T-helper lymphocytes in hereditary hemorrhagic telangiectasia

**DOI:** 10.3389/fimmu.2023.1321182

**Published:** 2023-12-08

**Authors:** Alexandre Guilhem, Marion Ciudad, Marie-Hélène Aubriot-Lorton, Hélène Greigert, Claudie Cladière, Vanessa Leguy-Seguin, Sylvain Audia, Maxime Samson, Bernard Bonnotte

**Affiliations:** ^1^ Service de Médecine Interne et Immunologie Clinique, Centre de compétence maladie de Rendu-Osler, Centre Hospitalo-Universitaire Dijon Bourgogne, Dijon, France; ^2^ Université de Bourgogne, INSERM, EFS BFC, UMR1098, RIGHT Interactions Greffon-Hôte-Tumeur/Ingénierie Cellulaire et Génique, Dijon, France; ^3^ Service d’Anatomie-Pathologie, Centre Hospitalo-Universitaire Dijon Bourgogne, Dijon, France

**Keywords:** hereditary hemorrhagic telangiectasia, effector memory T-helper lymphocytes, Th2, Th17, Treg, VEGF-A

## Abstract

Hereditary hemorrhagic telangiectasia (HHT) is a rare inherited disease due to heterozygous loss-of-function mutations on the BMP9/10 pathway (*ENG, ACVRL1* or *MADH4* mainly). HHT endothelial cells are prone to lose their quiescence, leading to progressive appearance of numerous telangiectases on skin and mucosa (complicated by epistaxis and anemia), and to larger arteriovenous malformations in lungs, liver and brain. HHT is also associated with T lymphocyte abnormalities, which are currently poorly understood. We quantified by flow-cytometry the main T lymphocyte circulating subsets in 40 HHT patients and 20 matched healthy controls. Immunostaining was done on 2 HHT skin telangiectases. Disruptions in T lymphocyte homeostasis was observed, characterized by increases in subsets known to promote angiogenesis: Th2 (1.38% vs 1.15%, p=0.021), Th17 (0.32% vs 0.22%, p=0.019 2) and Treg (4.94% vs 3.51%, p= 0.027). T angiogenic lymphocytes (Tang), defined as CD3+CD31+CXCR4+ T cells, were at similar levels in both groups, but the proportion of VEGF-A+ Tang after stimulation was higher in the HHT group compared to controls (68.2% vs 44.9%, p=0.012). The global HHT T lymphopenia predominantly affected the effector memory T-helper cells (200 vs 270 cells/mm^3^, p=0.017), and the lymphocytic infiltrate around HHT telangiectases consisted of memory T-helper cells. The Th17 circulating subset was positively correlated with the monthly epistaxis duration (r coefficient: +0,431, p=0.042), prospectively assessed. HHT T-helper lymphocytes are affected by several pro-angiogenic changes, potentially resulting from their recruitment by abnormal endothelial cells. They could constitute a biologically relevant source of VEGF-A and a valuable therapeutic target in HHT.

## Introduction

Hereditary hemorrhagic telangiectasia (HHT) is a vascular genetic disease with a prevalence estimated to be 1/6000 (1). This condition is due to heterozygous loss-of-function variants of three main genes (*ENG, ACVRL1, MADH4)*, all belonging to the BMP9/10 pathway that maintains endothelial cells in a quiescent and mature state (2). The pathway disruption alters several functions of endothelial cells, including migration against shear stress, regulation of proliferation, crosstalk with pericytes and response to Vascular Endothelial Growth Factor (VEGF) signaling (3). The resulting pathological angiogenesis is characterized by the formation of arteriovenous malformations (AVM), corresponding to direct connections between arterial and venous vessels without an intervening capillary bed. At a microvascular level, it explains the progressive appearance of multiple telangiectases on the skin (especially the hands and face) and on the mucous membranes (nose, mouth and digestive tract). The mucosal telangiectases are responsible for recurrent spontaneous epistaxis and more rarely gastro-intestinal bleeding, which can both lead to iron-deficiency anemia. Larger AVM’s can occur in the lungs, the liver and the central nervous system. They can lead to life-threatening events: cerebral abscess, high-output cardiac failure or cerebral hemorrhage (4).

Implication of T lymphocytes in HHT’s abnormal angiogenesis is suggested by their presence in the mononuclear infiltrate surrounding the HHT telangiectases in skin biopsies (5) and by their unexplained decrease in circulating blood of HHT patients (6). T lymphocytes do not strongly express HHT-related genes, but they have numerous interactions with endothelial cells during their transit from blood circulation to their target tissues. On one side, activated endothelial cells can act as a semi-professional antigen presenting cell (APC) by recruiting, activating and modulating the polarization of T lymphocytes (7–10). In return, T lymphocytes can enhance angiogenesis by the production of angiogenic factors, including VEGF-A (11). A T lymphocyte subset, recently identified with a CD3+CD31+CXCR4+ phenotype, has been shown to have multiple *in vitro* and *in vivo* pro-angiogenic capacities through VEGF-A, IL-8 and IL-17 production (12). This so-called “T angiogenic” (Tang) subset is altered in acquired vascular diseases (13) and auto-immune diseases with vascular involvement (14) but has never been studied in HHT.

To further explore the links between immunity and angiogenesis in HHT, we compared circulating lymphocyte subsets of HHT patients and matched healthy subjects. We studied tissue lymphocytes by immunostainings on skin biopsies of HHT telangiectases. We investigated correlations between immunological changes and epistaxis durations (prospectively collected), as well as other major features of HHT.

## Materials and methods

### Subject selection

Patients and healthy controls were prospectively recruited between June 2018 and February 2020 in the HHT Competence Center of the University Hospital of Dijon-Bourgogne (France).

Subjects under 18 years, those with active or recent (< 6 month) conditions known to activate or suppress the immune system (infection, surgery, pregnancy, solid cancer or lymphoma, auto-immune disease, immunosuppressant or systemic corticosteroid therapy) and those with treatment interfering with angiogenesis (bevacizumab, tranexamic acid, DPP-4 inhibitors, and beta-blockers) were excluded.

The diagnosis of HHT was based on at least 3 Curaçao criteria and was genetically confirmed for every selected patient. A thoracic computed tomography scan and an echographic liver assessment were systematically proposed to all patients, according to the French guidelines for HHT diagnosis and treatment. Cerebral or gastrointestinal tract investigations were proposed only according to the clinical context, including symptoms, clinical signs and personal or family histories. Patients having undergone a skin biopsy of telangiectases for a clinical purpose were identified during the screening process. Healthy controls (HC) were recruited among healthcare workers of the University Hospital of Dijon-Bourgogne (France) and their relatives with a ratio of 1 HC for 2 included patients, matched for age (+/- 6 years) and sex.

### Study design

Patients were included during their scheduled routine visit in the HHT center, after their medical visit. The Epistaxis Severity Score (ESS) was calculated at inclusion, and data concerning HHT characteristics (visible telangiectases, arteriovenous malformations, infectious history), cardiovascular risk factors (active tobacco smoking, treated HTA, dyslipidemia, diabetes mellitus, body mass index > 30) and treatments (including iron replacement) were collected. Blood samples were collected to perform routine blood tests according to hospital procedures (complete blood cell count, C-reactive protein, ferritin, creatinine), and PBMC’s were isolated to perform subsequent immunophenotyping. The duration and number of epistaxis episodes were recorded by the patients using epistaxis grids during the 3 months after inclusion. Concerning healthy subjects, a standard medical assessment (including a questionnaire on cardiovascular risk factors) was performed before blood sampling.

This research study was registered on clinicaltrials.gov with the number NCT03572556. The protocol was registered and approved by a French ethics committee (Comité de Protection des Personnes SUD-EST IV, ID-RCB: 2017-A03280-53 - CPP: 17/086). All subjects gave written informed consent before their inclusion, in accordance with the Declaration of Helsinki.

### Immunophenotyping

PBMC’s were isolated by Ficoll density gradient within a few hours after blood collection on lithium heparinate tubes. Immunophenotyping was performed according to the latest guidelines (15).

Intracellular cytokine production was measured after stimulation with phorbol-12-myristate-13-acetate (PMA, 100 ng/mL, Sigma-Aldrich) and ionomycin (1µg/mL, Sigma-Aldrich), for 4 hours, in the presence of brefeldin A (GolgiPlug, 0.1%, BD Biosciences). The following monoclonal antibodies were used: Brilliant Violet (BV) 510 anti-CD3, BV421 anti-CD4, Phycoerythrin (PE) anti-IL-8, BV421 anti-CXCR4, Fluorescéine-5-isothiocyanate (FITC) anti-CD31, Alexa Fluor 488 anti-FoxP3, Phycoerythrin Cyanin5 (PECy5) anti-CD4/PE anti-CD25 (from BioLegend); FITC anti-CD8, PeCy7 anti-CD8, Allophycocyanin (APC) anti-CD4, PeCy7 anti-CD4, FITC anti-IL-4, APC anti-IFN-γ, PE anti-IL-17, PE anti-CD19, PE anti-CCR7, APCef780 anti-CD45RA (from eBioscience); APC anti-VEGF-A (from R&D systems). Data were acquired on BD Biosciences LSRII cytometer and analyzed with FlowJo^®^ v10 software (BD Biosciences).

### Histological analyses of skin telangiectases

Two HHT patients with genetic confirmation had undergone skin biopsy of hand telangiectases for clinical purposes, followed by a standard Formalin-Fixed Paraffin-Embedded procedure. The remaining material was used to cut 4 µm sections for conventional hematoxylin-eosin-saffron (HES) staining and immunohistochemistry. Deparaffinization and immunolabeling were performed with a fully automatic system Dako Omnius, using the following antibodies (all from Dako): anti-CD3 (polyclonal rabbit anti human), anti-CD4 (AB12 clone), anti-CD8 (C8/114 clone) and anti-CD45RO (VCHL1 clone).

### Statistical analysis

Given the low number of subjects, we only used non-parametric tests. Comparisons between groups were performed using Mann–Whitney U-tests (for two groups) or Kruskal-Wallis tests (for three groups). Correlations between numerical variables were analyzed with the Spearman’s rank test.

A p-value <0.05 was considered statistically significant. In case of multiple tests (> 5) on the same variable, we applied a Bonferroni correction with an alpha value set to 0.05.

All the analyses were performed with Graphpad Prism version 9.0.0.

## Results

### Main characteristics of the study population

Forty patients (20 men and 20 women) with a median age of 52 years (min-max: 19-74 years) were recruited along with 20 age- and sex-matched healthy controls (HC). Their main characteristics are detailed in **Table 1**.

**Table 1:** Main characteristics of the study population.

	HHT (n=40)	Controls (n=20)	
Age (median (min-max))	52.1 (19-74.3)	50.7 (24.6-80.2)	p = 0,947
Sex (males, %)	20 (50%)	10 (50%)	p > 0.999
Evitable cardiovascular risk factors* (median (min-max))	1 (0-3)	0 (0-3)	p = 0,160
Blood cell count (median (min-max)):			
Hemoglobin (g/dl)	14.2 (9.3-17.1)	14.9 (12.5-15.9)	p = 0.126
Mean corpuscular volume (fl)	88 (76-99)	88 (78-93)	p = 0.972
Platelets (G/l)	262 (175-631)	270 (158-332)	p = 0.800
Neutrophils (G/l)	3.79 (1.60-8.52)	3.09 (1.96-7.25)	p = 0.102
Lymphocytes (G/l)	**1.54 (0.70-3.09)**	**2.31 (1.00-4.00)**	**p < 0,001**
Monocytes (G/l)	0.47 (0.31-0.96)	0.47 (0.29-1.24)	p = 0.997
Mutation (n, (%)):			
*ENG*	17 (43%)		
*ACVRL1*	22 (55%)		
*SMAD4*	1 (3%)		
Mean monthly duration of epistaxis (in minutes/month) (median (min-max))	18 (0-585)		
Epistaxis Severity Score (median (min-max))	1.72 (0-5.47)		
Visible telangiectases (n, (%))			
<5	10 (25%)		
5-30	14 (35%)		
>30	16 (40%)		
Arteriovenous malformations (n/nt, (%))			
Lungs	20/40 (50%)		
Digestive tract	6/10 (60%)		
Liver	10/40 (25%)		
Brain	9/25 (36%)		
History of severe infection (n, (%))	6/40 (15%)		
Iron replacement therapy (n, (%))			
Any	25 (62%)		
Oral supplementation	8 (20%)		
IV supplementation and/or blood transfusion~	7 (18%)		

n: number of subjects.

nt: number of tested subjects.

IV: Intra-Venous.

*: active tobacco smoking, treated HTA, dyslipidemia, diabetes mellitus, body mass index > 30.

~: at least once during the last year.

The values with a statistical significativity have been highlighted in bold.

The HHT group was well-balanced between *ACVRL1* (55%) and *ENG* (43%) mutations, and only one patient was *SMAD4* mutated. Patients had generally low-to-moderate grade epistaxis (median ESS: 1.72, min-max: 0-5.47) and were mostly independent of any type of iron supplementation (62% of the cohort). The duration of epistaxis was highly variable, from 0 to 585 min/month, with 15 patients at less than 10 min/month and 8 patients at more than 100 min/month. Thirty-three patients (83%) had at least one visceral AVM, mainly in the lungs (20 cases) and the liver (10 cases). Six patients (15% of the cohort) had a history of severe infection, including 2 cerebral abscesses (*Streptococcus constellatus* for both, associated with anaerobic bacteria for one), 1 osteomyelitis, 1 salpingitis with abscess, 1 *Clostridium difficile* acute colitis and 1 infectious pleuritis.

The complete blood cell count showed a decreased level of total circulating lymphocytes in the HHT group (median HHT vs HC: 1.54 vs 2.31 G/l; p<0.001) with comparable levels for the other parameters of the full blood count.

### Study of T cell polarization

**Figure 1:**
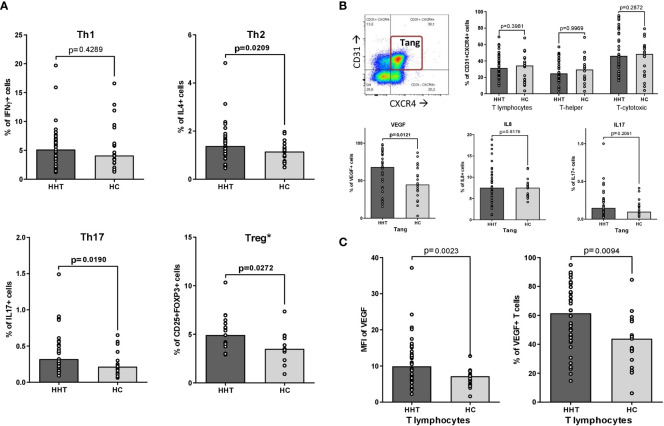
Th1, Th2, Th17, Treg and Tang polarizations. Comparisons between the HHT group (HHT: dark gray) and the healthy control group (HC: light gray): **(A)** T-helper polarizations after PMA/ionomycin stimulation: • Th1 (CD3+CD4+interferon γ+IL-17-, after PMA/ionomycin stimulation). • Th2 (CD3+CD4+IL-4+, after PMA/ionomycin stimulation). • Th17 (CD3+CD4+interferon γ-IL-17+, after PMA/ionomycin stimulation). • Treg (CD3+CD4+CD25+FOXP3+)*. **(B)** T angiogenic lymphocytes (Tang: CD3+CD31+CXCR4+): • A representative dot plot illustrating the gating strategy, according to ref (12). • Percentage of Tang in total T lymphocytes, T-helper lymphocytes and T-cytotoxic lymphocytes. • Intracytoplasmic production of pro-angiogenic cytokines by Tang after PMA/ionomycin stimulation: VEGF-A, IL-8 and IL-17. **(C)** VEGF-A synthesis by T lymphocytes after PMA/ionomycin stimulation: • Percentage of T lymphocytes positive for intracytoplasmic VEGF-A. • Mean Fluorescence Intensity (MFI) of intracytoplasmic VEGF-A in total T lymphocytes. Results are presented by bar charts indicating the median and aligned individual values. Comparisons were performed using the Mann-Whitney test. P-values below 0.05 are considered significant and shown in bold. * only on 19 HHT and 13 HC subjects.

As shown in **Figure 1A**, the proportions of Th2, Th17 and Treg lymphocytes were significantly increased in HHT patients (median: 1.38 vs 1.15%, p=0.021; 0.32 vs 0.22%, p=0.019 and 4.94 vs 3.51%, p= 0.027, respectively), with no change in the Th1 subset.

The assessment of Tang lymphocytes is presented in **Figure 1B**. There was no significant difference in the percentage of Tang in the total T lymphocyte population between HHT and HC, as well as in the T-helper and T-cytotoxic subsets. After stimulation, the percentage of VEGF-A+ Tang was increased in the HHT group (median: 68.2 vs 44.9%, p=0.012) while the levels of IL-8 and IL-17 producing Tang remain stable.

The same phenomenon was observed at the level of the whole T lymphocyte population (**Figure 1C**): the percentage of VEGF-A+ cells and the MFI levels were significantly higher in the HHT T lymphocytes compared to HC (respectively 61.5 vs 43.8, p=0.009 and 9.92 vs 7.18, p=0,002).

### Main circulating and peri-vascular lymphocyte subsets

**Figure 2:**
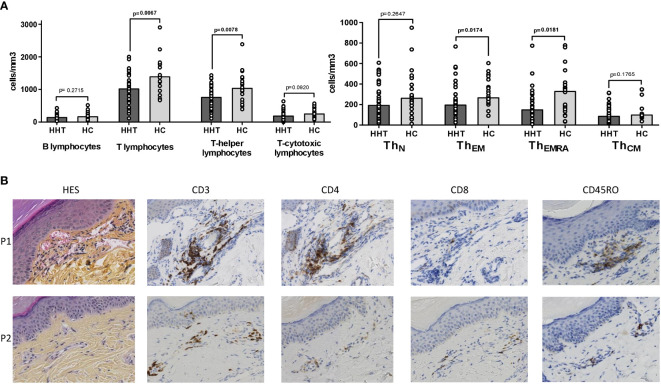
Circulating and peri-vascular lymphocyte subsets. **(A)** Comparisons between the HHT group (HHT: dark gray) and the healthy control group (HC: light gray) on: • Lymphocyte subsets: B (CD19+), T (CD3+), T-helper (CD3+CD4+) and T-cytotoxic (CD3+CD8+). • T-helper subsets: naïve (T_N_: CCR7+CD45RA+), effector memory (T_EM_: CCR7-CD45RA-), effector memory RA+ (T_EMRA_: CCR7-CD45RA+), central memory (T_CM_: CCR7+CD45RA-). **(B)** Skin biopsies of hand telangiectases from 2 HHT patients (P1: 69 years old male with *ACVRL1* mutation, P2: 40 years old male with *ACVRL1* mutation): conventional hematoxylin-eosin-saffron (HES) staining, immunohistochemistry staining with anti-CD3, anti-CD4, anti-CD8 and anti-CD45RO antibodies. Results are presented by bar charts indicating the median and aligned individual values. Comparisons were performed using the Mann-Whitney test. P-values below 0.05 are considered significant and shown in bold.

The HHT lymphocyte decrease significantly affected the T-helper subset (median: 1031 vs 1405 cells/mm^3^, p=0.0067) but spared B and T-cytotoxic lymphocytes, as visible in **Figure 2A**.

Among the T-helper subsets (**Figure 2B**), the decrease was significant for the Th_EM_ and Th_EMRA_ populations (median: 200 vs 270 cells/mm^3^, p=0.017 and 153 vs 332 cells/mm^3^, p=0.018, respectively), whereas the naïve and central memory subsets were similar in HHT and HC groups.

**Figure 2C** shows histological sections of cutaneous telangiectases of two patients. The first patient (P1) is a 69-year-old male carrying an *ACVRL1* mutation, and the second patient (P2) is a 40-year-old male also with an *ACVRL1* mutation. The HES staining revealed dilated capillaries of the superficial dermis with a moderate perivascular mononuclear infiltrate. This infiltrate consisted mainly of memory T-helper lymphocytes characterized by CD3, CD4 and CD45RO expression on immunostaining. This infiltrate was more evident in P1 than in P2.

### Relations between main immunological changes and features of HHT

As shown in **Tables 2A–C**, there was no association between the T lymphocyte changes and the mutated gene (*ENG* or *ACVRL1*), the history of severe infection or the level of iron requirement.

**Table 2:** Relationships between main HHT characteristics and immunological changes (median (min-max)).

	Th2 (%)	Th17 (%)	Treg^1^ (%)	T_VEGF_ (%)	Tang _VEGF_ (%)	Th_EM_ (/mm^3^)	Th_EMRA_ (/mm^3)^
a) Genotype							
*ENG*	1.25 (0.46-4.83)	0.42 (0.13-1.49)	4.06 (2.92-6.97)	68.2 (14.7-88.8)	72.8 (19.0-78.3)	181 (55-568)	215 (38-774)
*ACVRL1*	1.52 (0.61-2.38)	0.28 (0.09-0.91)	5.52 (3.02-10.3)	54.1 (19.5-94.9)	60.1 (15.4-83.0)	200 (26-765)	151 (8-503)
p-value*	0,910	1,000	0,469	1,000	1,000	1,000	1,000
b) History of severe infection							
Yes	1.41 (0.85-1.66)	0.35 (0.22-0.88)	4.01 (2.92-6.95)	69.5 (46.8-94.9)	76.9 (52.2-94.4)	371 (74-765)	143 (14-503)
No	1.38 (0.46-4.83)	0.32 (0.09-1.49)	5.16 (2.99-10.3)	57.1 (14.7-88.8)	64.5 (15.4-81.9)	186 (26-559)	162 (8-774)
p-value*	1,000	1,000	1,000	1,000	1,000	0,497	1,000
c) Iron requirements							
No iron	1.27 (0.61-2.38)	0.26 (0.09-0.88)	5.16 (2.99-10.3)	56.6 (23.0-94.9)	64.5 (15.4-98.4)	225 (55-765)	174 (38-503)
Oral iron	1.42 (0.46-3.13)	0.39 (0.09-0.86)	3.02 (2.92-6.11)	65.9 (19.5-86.1)	77.4 (15.4-92.1)	201 (121-244)	127 (43-774)
IV iron or blood transfusion	1.48 (0.54-4.83)	0.52 (0.28-1.49)	NVA	69.5 (14.7-88.8)	71.2 (19.0-97.3)	116 (26-300)	59 (8-338)
p-value~	1,000	0,259	1,000	1,000	1,000	0,336	1,000
d) Monthly Epistaxis Duration							
r coefficient (Spearman)	0,165	**0,431**	-0,464	-0,208	-0,223	-0,292	-0,268
p-value*	1,000	**0,042**	0,322	1,000	1,000	0,476	0,658
e) Evitable cardiovascular risk factors^							
Any	1,48	0,29	5,66	66,8	71,1	225	130
At least one	1,27	0,41	4,12	54,2	59,5	159	220
p-value*	1	1	1	1	1	0,42	0,91

T_VEGF_, T VEGF-A+ lymphocytes.

Tang _VEGF_, T angiogenic VEGF-A+ lymphocytes.

Th_EM_, T-helper effector memory lymphocytes.

Th_EMRA_, T-helper effector memory RA+ lymphocytes.

IV, Intra-Venous.

NVA, no value available.

^1^: only on 19 HHT subjects.

^: active tobacco smoking, treated HTA, dyslipidemia, diabetes mellitus, body mass index > 30.

*: Mann-Whitney p-value with Bonferroni correction.

~: Kruskal-Wallis p-value with Bonferroni correction.

The values with a statistical significativity have been highlighted in bold.

The mean monthly epistaxis duration, measured prospectively during 3 months after inclusion, was positively correlated with the level of Th17 polarization (r coefficient: +0,431, p=0.042), as shown in **Table 2D**.

In **Table 2E**, we found no significant difference in immunological changes between HHT patients with no cardiovascular risk factors and those with at least one.

## Discussion

We report here for the first time that HHT is associated with an increase in T-helper subsets capable of promoting angiogenesis. This specific pro-angiogenic shift involves the percentage of Th2, Th17, Treg and the VEGF-A production by Tang and total T lymphocytes.

The Th2 and Th17 lymphocytes are known to promote angiogenesis *in vitro* and in mouse models (11, 16). In humans, the Th17 subset plays a pro-angiogenic role in ocular neo-vascular diseases (17) and cancer (18). Th2 lymphocytes induce pathological angiogenesis in asthma, via IL-25 production (19). The effect of Treg lymphocytes on angiogenesis seems to be context and tissue dependent, but this subset is generally considered to be a promoter of angiogenesis (20).

VEGF-A is a fundamental regulator of angiogenesis by inducing endothelial proliferation and migration, increased vascular permeability and destabilization of the local extracellular matrix (21, 22). T-helper and T-cytotoxic lymphocytes have been known for a long time to secrete VEGF-A at a bioactive concentration, a phenomenon implicated in tumor angiogenesis (23) and diabetic retinopathy (24). The VEGF pathway is demonstrated to be crucial in HHT since its blockade by monoclonal antibodies or tyrosine kinase inhibitors significantly improve epistaxis, digestive bleeding, and high-output heart failure (25, 26). However, we still poorly understand to date how alteration of the BMP9/10 pathway increases the VEGF-A activity (27). Our results suggest that T-helper lymphocytes could participate in the formation of the focally dilated and frail capillaries observed in HHT, possibly by local and transient production of angiogenic cytokines including VEGF.

This pro-angiogenic T-helper shift seems to result from abnormal interactions between HHT endothelial cells and lymphocytes. This hypothesis is suggested by the presence of memory T-helper cells in the perivascular infiltrate surrounding telangiectases. This possibly results from their recruitment by activated endothelial cells, which are semi-professional antigen-presenting cells (APC) with important immunomodulatory capacities (9, 28). They can produce IL-6 and promote the Th17/Treg subsets (10), a phenomenon well-described in allograft rejection (7), which may occur in HHT due to the defective endothelial quiescence resulting from disruption of the BMP9/10 pathway (29). The APC role of endothelial cells is limited to memory lymphocytes because they do not express CD80-CD86, which is required to interact with naive lymphocytes (8). Therefore, the decrease in circulating Th_EM_ and Th_EMRA_ reinforces the hypothesis of pathological recruitment of T lymphocytes by HHT endothelial cells. Previously, it was reported that the decrease in T cells in HHT mainly affected naive cells but used a confusing phenotypic definition based only on CD45RA expression (6).

By studying the relationship between the different T cell changes and the main HHT features, the only significant correlation was between the Th17 percentage and the mean monthly duration of epistaxis, prospectively assessed during 3 months after inclusion. Interestingly, the Th17 subset exerts its immunological functions at the body’s barrier sites (30) which include the skin and nasal/digestive mucosal membranes, the same anatomical areas affected by HHT telangiectasia. Moreover, Th17 lymphocytes are capable of producing potent angiogenic cytokines, including IL-17, PlGF and IL-22 (16, 31, 32). In the context of HHT, this could result in a pro-angiogenic amplification loop: proliferating endothelial cells could induce recruitment and polarization of TH17 lymphocytes, which in return would activate and destabilize endothelial cells by secreting angiogenic cytokines. The Th17 subset could be investigated as a potential biomarker of HHT severity. It could also be a valuable therapeutic target for drug repurposing, as monoclonal antibodies inhibiting the Th17 pathway are already available. The T cell changes do not seem to be driven by atherosclerosis as a confounding factor, since we found no association with cardiovascular risk factors.

Our study suffers from several limitations. First, the increase of VEGF-A production has been assessed after PMA/ionomycin stimulation, and its extracellular secretion have not been quantified. The characterization of peri-telangiectasic lymphocytes should be interpreted with caution, given the limited number of immunostainings performed on only two biopsies. *In vitro* functional tests assessing the proliferation and activation capacities of lymphocytes and EC could have provided a better understanding of the underlying mechanisms. The Th9 and Th22 subsets, more recently described, could also be altered in HHT but were not studied here. All these points will be addressed in future studies.

Detailed phenotypic descriptions of T lymphocytes in HHT revealed pro-angiogenic changes including higher levels of Th2, Th17, Treg and VEGF-A production capacities. Abnormal recruitment of memory T lymphocytes by pathologically activated endothelial cells is suspected by observations on perivascular and circulating lymphocytes. The Th17 subset seems of particular interest given its correlations with epistaxis duration. A better understanding of the immunological changes associated with HTT may offer new therapeutic targets for HHT patients in the future.

## Data availability statement

The raw data supporting the conclusions of this article will be made available by the authors, without undue reservation.

## Ethics statement

The studies involving humans were approved by Comité de Protection des Personnes SUD-EST IV, ID-RCB: 2017-A03280-53 - CPP: 17/086. The studies were conducted in accordance with the local legislation and institutional requirements. The participants provided their written informed consent to participate in this study.

## Author contributions

AG: Conceptualization, Data curation, Formal analysis, Funding acquisition, Methodology, Writing – original draft, Writing – review & editing. MC: Conceptualization, Data curation, Formal analysis, Project administration, Validation, Writing – review & editing. M-HA-L: Resources, Validation, Writing – review & editing. HG: Validation, Writing – review & editing. CC: Data curation, Formal analysis, Validation, Visualization, Writing – review & editing. VL-S: Conceptualization, Investigation, Writing – review & editing. SA: Conceptualization, Investigation, Resources, Writing – review & editing. MS: Conceptualization, Investigation, Resources, Validation, Writing – review & editing. BB: Conceptualization, Formal analysis, Funding acquisition, Investigation, Project administration, Resources, Supervision, Validation, Writing – review & editing.
